# Workplace mentoring of residents in generic competencies by an independent coach

**DOI:** 10.1007/s40037-018-0452-7

**Published:** 2018-09-05

**Authors:** Jos A. Stigt, Janine H. Koele, Paul L. P. Brand, Debbie A. C. Jaarsma, Irene A. Slootweg

**Affiliations:** 1 Department of Pulmonology, Isala Hospital, Zwolle, The Netherlands; 2 Isala Women’s and Children’s Hospital, Zwolle, The Netherlands; 3 Center for Education Development and Research in Health Professions, University of Groningen and University Medical Center Groningen, Groningen, The Netherlands; 4 Department of Public Health and Primary Care, Leiden University Medical Center, Leiden, The Netherlands; 5 UMCG Postgraduate School of Medicine, University of Groningen and University Medical Center Groningen, Groningen, The Netherlands

**Keywords:** Mentoring, Education/teaching, Communication skills, Direct observation, Feedback

## Abstract

**Introduction:**

During postgraduate education in pulmonology, supervisors are responsible for training residents in generic competencies such as communication, professionalism and collaboration, but their focus commonly lies more on medical-technical competencies. As an alternative approach to supporting residents to develop generic skills, we developed a personal mentoring program with a non-medical professional as mentor. In this study, the residents’ experiences with the mentoring program were evaluated.

**Methods:**

After an introductory session in which individual learning goals were established, pulmonology residents received at least six, 60–90-minute, individual, mentoring sessions largely consisting of feedback after being observed during daily clinical activities, over a period of 9 months. The residents’ experiences with mentoring were explored through in-depth interviews followed by a qualitative content analysis.

**Results:**

From March to November 2016, ten residents in pulmonology completed the program. Despite initial scepticism, mentoring encouraged residents to reflect deeply on their professional interactions. This caused an increased awareness of the effects of their communication and behaviour on patients. Experimenting with communication and different behaviours in subsequent interactions felt rewarding and contributed to further development, resulting in increased self-confidence and job satisfaction.

**Discussion:**

Mentoring residents by non-medical coaching was associated with improved residents’ proficiency in generic competencies.

## Introduction

Workplace learning is the mainstay of postgraduate medical education applying to medical skills as well as to generic skills. In daily practice, residents develop generic competencies as they receive continuous informal feedback when communicating with patients, other residents, senior doctors, allied healthcare professionals and nurses [1].

Clinical supervisors help residents to develop skills in these competencies. This can be practised by direct observation and feedback but this is not structurally implemented everywhere in postgraduate training [2, 3]. Supervisors tend to focus their feedback on diagnostic and technical skills and provide relatively little feedback on generic competencies such as communication, professionalism and collaboration [4]. The quality of feedback on generic competencies is often limited [5].

Other factors may limit the opportunities for residents to deliberately practise generic competencies, such as lack of supervision in busy medical practices and the supervisor’s dual and sometimes conflicting roles as teacher and mentor [6–8].

The question arises whether supervisors are the most appropriate teachers for all residents and in all circumstances to support their development in generic competencies. To help residents to improve generic competencies, we developed a mentoring program with a non-medical coach observing residents in the workplace while performing daily clinical tasks and providing structured feedback. This study explored pulmonology residents’ experiences with this mentoring program.

## Methods

### Context, study population and analysis

Residents working at the Department of Pulmonology of Isala, a large teaching hospital in Zwolle, the Netherlands, participated in the mentoring program. Their experiences with mentoring were evaluated by content analysis of semi-structured interviews carried out by an independent senior qualitative researcher after completion of the mentoring program and performed according to COREQ guidance principles.

### The mentoring program

The mentoring program was designed and performed by a single coach trained in Facility Management, Leadership Development and Organisational Science, with 10 years’ experience in counselling, management coaching and interim management in both commercial and healthcare organisations.

During mentoring, residents received applicable tips to improve their communication and collaboration skills. They were encouraged to reflect on their thoughts (for example their assumptions) and behaviour towards others, and to analyze this in order to develop more self-insight. They were challenged to vary and modulate their reactions to patients’ verbal and non-verbal communication. By try outs, they learned to anticipate and prepare subsequent interactions.

The program started with a plenary meeting for all residents in which the aims and the content of the program were discussed. Subsequently, each resident participated in six individual 60–90-minute sessions with the mentor, over a timeframe of 9 months. During the first individual session, residents’ personal norms and values and motivations, background, learning goals and needs and expectations regarding the mentoring program were discussed.

Subsequent individual sessions involved a brief preparation in which the resident discussed learning objectives with the mentor. The mentor observed the resident while performing daily clinical tasks (ward rounds, outpatient clinical visits, procedure room or patient handover), followed by a structured reflection and feedback session. Prior to each observation, the resident introduced the mentor to the patient, informed him/her about the mentoring program and asked the patient for consent regarding the presence of the mentor. During each encounter, the mentor did not intervene in any way. Individual sessions after each observed professional task also allowed time for mentor/mentee conversations on issues unrelated to the working context, the contents of which varied, depending on the resident’s personal needs and development phase.

The study was approved by the Netherlands Association for Medical Education (NVMO) ethical review board (file number 606). Written informed consent was obtained from all participants.

## Results

From March to November 2016, ten residents, in all phases of their training, completed the mentoring program. In Table 1 quotes are presented illustrating the participants’ experiences with mentoring.

**Table 1:** Quotes from the residents illustrating their experience with mentoring

**Communication**
*R2: The mentor pointed out that there is often more behind the emotions displayed by patients. When a patient gets angry, it is usually not because he just likes to shout at doctors. Therefore, it is better to identify the cause of the patient’s problem and whether it can be solved*
*R4: We discussed my interactions with patients prior to my encounters with them. After I had formulated my personal preferred approach, the mentor provided me with alternatives. I found a compromise and I got feedback afterwards. So, I got immediate feedback on my performance and we discussed alternative approaches to improve subsequent interactions with patients*
*R10: A patient’s spouse communicated in an aggressive and dominant way. When evaluating this consultation, the coach challenged me to re-label the spouse’s behaviour. I postulated fear as the reason for the displayed behaviour. On the next occasion, I confronted the spouse with this conclusion. On subsequent visits the atmosphere had clearly improved*
**Collaboration**
*R1: The mentor also made me aware of my own part in the interaction with the supervisors and provided me with tools to participate constructively in conversations. I found that this worked for me and I continued doing so*
*R8: I felt that I had a pleasant way of collaborating with colleagues, but I became aware that I can be quite compelling, which means that I can sometimes dominate others. This made me realise I was less approachable than I thought. The mentor confronted me with this and I had to admit that this behaviour sometimes provokes undesirable reactions*
*R4: As a starting resident you must find your role in the collaboration with nursing staff. They expect you to possess a certain know-how and to take the initiative by letting them know how you prefer to work. The mentor provided suggestions on how to tackle this without having to leave my comfort zone*
**Professionalism**
*R5: I have to watch out not to get overwrought … I already knew that, but I did not do anything about it. The mentor helped me better recognize the signals and how to react to them. Otherwise things could have gone wrong, for sure*
*R7: Due to personal circumstances, my correspondence with general practitioners and medical record administration had fallen far behind schedule. My program director informed me that it had been decided to prolong my medical training in order for me to work on this. The mentor helped me through this difficult phase by helping me vent my emotions and frustrations and motivating me throughout the rest of medical training. The mentor helped me to abandon my preconceived ideas about the program director and to approach him objectively. I was advised not to postpone problems, but to try to solve them in their infancy. After analyzing the problem, the mentor helped me write an action plan with detailed solutions. I could not have done this on my own*
*R9: Assuming a forced and detached doctor’s role was my unconscious way of hiding a lack of self-confidence in the beginning. A combination of feeling uncertain and expressing myself to patients not clearly, caused me to have difficulties in my encounters with patients as well as their families. I developed a more decisive type of communication with patients*

### Communication

All residents reported that the mentoring program had the most striking effects on their communication skills, not only with patients, but also with supervisors, colleagues and other healthcare professionals. They learnt to practise their communication techniques, try out new approaches, and apply structure to professional conversations. They also experienced an increased awareness of the effects of their communication and how it affected others.

The residents perceived that their communication with patients had improved as a result of feedback and better preparation. In addition to discussing medical content, they felt more comfortable in expressing empathy with the patient’s context.

### Collaboration

The mentoring program increased residents’ consciousness regarding the importance of effective collaboration and their insight into their personal motivations, norms and values with respect to collaboration, whatever the stage of training. The mentoring sessions enabled them to discuss their emotions with supervisors, colleagues and other healthcare professionals and also respond to these emotions. This improved their ability to engage in constructive dialogue and to reach agreement on collaboration. Changes perceived in this domain were experienced as having a positive influence on the learning environment.

### Professionalism

The residents reported that mentoring improved their ability to identify and maintain boundaries in work-life balance. They developed a better understanding of their role in relation to their fellow residents, and how to assert their role within the group. The residents took more responsibility for optimizing work processes and educational activities. Mentoring helped, especially the younger residents, to increase self-confidence and assume their new role as doctors.

### Features of mentoring

The residents experienced mentoring as safe, credible and engaging. They characterized the mentoring as very personal, inevitable and with guaranteed feedback. They appreciated the strict focus on generic competences contrasting to the feedback they received from supervisors, which was largely based on medical technical aspects. Residents appreciated that—in contrast to their previous experiences with observations made by supervisors—they did not feel as if they were being assessed during the mentoring program.

## Discussion

A mentoring program for generic competencies, added to pulmonology residency training, had a positive impact on the individual participants. Although the most profound results were reported in the domain of communication, residents also reported progress in professionalism and collaboration, independent of their phase of training.

### The resident at the workplace

The perceived benefits of mentoring can most likely be explained by some key features of deliberate practice that were used to improve performance. The residents formulated learning goals in advance with the mentor. Repeated observations of learning activities with immediate feedback provided by the mentor, allowed the residents to try out newly learnt communication and collaboration skills [9]. A body of evidence supports the usefulness of immediate structured feedback for learning in the clinical workplace, in contrast to more traditional teacher-centred programs [10].

At the start of the mentoring program, some residents expressed scepticism on the usefulness of the program, which appeared to be based on their previous experiences with teaching communication and collaboration skills during preclinical training. This contrasted with the enthusiasm they expressed after completing the mentoring program and suggests initial unconscious incompetence in communication, collaboration and professionalism skills, and a limited trust in their ability to learn these skills.

### The supervisor as clinician educator

Isolated supervisor training, a competency framework and assessment instruments are apparently not enough for optimal clinical learning [5]. The mentoring program created supervisors’ awareness of their role in coaching generic competencies but in practice they combine medical assessment and feedback. Since receptivity to feedback is disturbed by assessment, the combination is considered to be an ‘uneasy alliance’ [7]. Our residents reported that they felt they were being assessed on their medical competence whenever they were being observed by supervisors, even if supervisor and resident agreed beforehand that the observation was to be focused on generic competencies. This contrasted with their experiences with the non-medical mentor who only provided formative feedback.

### The mentor at the workplace

Mentoring of communication and behavioural skills performed by a non-medical professional worked well in our study. In comparison with clinical supervisors, the non-medical mentor provided more in-depth analysis and feedback about observed encounters with patients and colleagues. Unravelling the underlying causes of observed behaviour or communication appeared to be the trigger of residents’ behavioural changes and improvement of skills. In addition to a dialogue centred around deeper motivations, the mentor discussed follow-up measures and provided further help and guidance which are considered elements of ideal positive role modelling [8].

The efficacy of mentoring is likely to rely on the quality of the relationship between the mentor and the resident, which should be focused on the resident and provide guaranteed emotional security [11]. The personalized and demand-driven approach used in this study proved suitable for building effective mentoring relationships and argues against uniformity in the way mentoring is performed [12].

The prolonged relationship with the mentor—lasting for up to 9 months—with repeated encounters created a feeling of relatedness, autonomy and competence in residents [10].

## Limitations

This study was conducted in a relatively small group of residents in a single medical specialism and by one mentor. Currently, research is in progress to study the reproducibility of the success of this approach in larger groups and other disciplines.

## Conclusion

Despite initial scepticism, all residents in this study experienced increased self-efficacy in generic competencies through mentoring provided by a non-medical professional, whatever their phase of training.
